# 4E-BP1–dependent translation in microglia controls mechanical hypersensitivity in male and female mice

**DOI:** 10.1172/JCI180190

**Published:** 2025-06-02

**Authors:** Kevin C. Lister, Calvin Wong, Weihua Cai, Sonali Uttam, Patricia Stecum, Rose Rodrigues, Mehdi Hooshmandi, Nicole Brown, Jonathan Fan, Noe Francois-Saint-Cyr, Shannon Tansley, Volodya Hovhannisyan, Diana Tavares-Ferreira, Nikhil Nageshwar Inturi, Khadijah Mazhar, Alain Pacis, Jieyi Yang, Alfredo Ribeiro-da-Silva, Christos G. Gkogkas, Theodore J. Price, Jeffrey S. Mogil, Arkady Khoutorsky

**Affiliations:** 1 Department of Anesthesia and; 2 Department of Pharmacology and Therapeutics, McGill University, Montreal, Quebec, Canada.; 3 Department of Neuroscience and Center for Advanced Pain Studies, University of Texas at Dallas, Dallas, Texas, USA.; 4 Canadian Centre for Computational Genomics, McGill Genome Centre, Montreal, Quebec, Canada.; 5 Department of Anatomy and Cell Biology and; 6 Alan Edwards Centre for Research on Pain, McGill University, Montreal, Quebec, Canada.; 7 Foundation for Research and Technology–Hellas, Heraklion, Greece.; 8 Department of Psychology, Faculty of Science and; 9 Faculty of Dental Medicine and Oral Health Sciences, McGill University, Montreal, Quebec, Canada.

**Keywords:** Cell biology, Neuroscience, Pain

## Abstract

Spinal microglia play a pivotal role in the development of neuropathic pain. Peripheral nerve injury induces changes in the transcriptional profile of microglia, including increased expression of components of the translational machinery. Whether microglial protein synthesis is stimulated following nerve injury and has a functional role in mediating pain hypersensitivity is unknown. Here, we show that nascent protein synthesis is upregulated in spinal microglia following peripheral nerve injury in both male and female mice. Stimulating mRNA translation in microglia by selectively ablating the translational repressor eukaryotic initiation factor 4E–binding protein 1 (4E-BP1) promoted the transition of microglia to a reactive state and induced mechanical hypersensitivity in both sexes, whereas spontaneous pain was increased only in males. Conversely, inhibiting microglial translation by expressing a mutant form of 4E-BP1 in microglia attenuated their activation following peripheral nerve injury and alleviated neuropathic pain in both sexes. Thus, stimulating 4E-BP1–dependent translation promotes microglial reactivity and mechanical hypersensitivity, whereas inhibiting it alleviates neuropathic pain.

## Abstract 2

Peripheral nerve injury triggers an increase in translation within spinal microglia, leading to their activation and the development of mechanical hypersensitivity.

## Graphical abstract

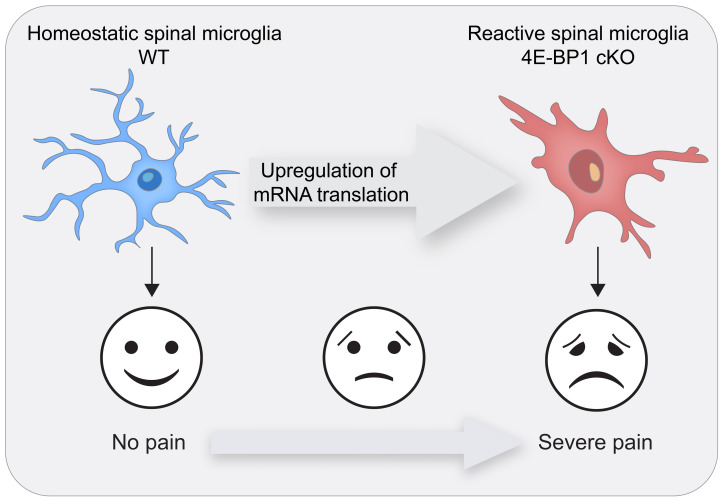

## Introduction

Peripheral nerve injury may lead to neuropathic pain, a prevalent and debilitating chronic pain condition. Nerve injury–induced proliferation and activation of microglia in the spinal cord is one of the key processes mediating the development of neuropathic pain (1–4). Activated microglia promote pain hypersensitivity through various mechanisms, including (i) enhancement of excitatory synaptic transmission via release of TNF, IL-1β, IL-6, PGE_2_, and ATP (5–8); and (ii) reduction in inhibitory tone via suppression of inhibitory synaptic transmission by proinflammatory cytokines (8, 9) and BDNF-mediated downregulation of K^+^-Cl^–^ cotransporter [KCC2] in neurons (10, 11), as well as via engulfment of inhibitory synapses (12, 13) and components of the extracellular matrix, such as perineuronal nets (14). The critical role of microglia in neuropathic pain is sexually dimorphic (15, 16). Whereas both sexes exhibit microglial proliferation and activation in response to peripheral nerve injury, the functional role of microglia in mediating neuropathic pain is predominantly observed in male animals (2, 17).

Stimulation of microglia in response to peripheral nerve injury is mediated via the release of proteases (18, 19) and bioactive molecules (2) from damaged or hyperactive sensory neurons in the spinal cord dorsal horn. These molecules bind to and activate their corresponding receptors on microglia (e.g., CSF1-CSF1R, ATP-P2X4R/P2Y12R, and CX3CL1-CX3CR1) (3, 20–22), triggering the downstream activation of intracellular signaling pathways such as p38, ERK1/2, and AKT (23–28) and eliciting changes in gene expression. Numerous studies have focused on transcriptional changes in microglia as an important mechanism to modulate their functional state. These studies have also revealed that peripheral nerve injury induces transcriptional upregulation of genes encoding for components of translational machinery, including the small and large ribosomal subunits and translational factors (29, 30). Furthermore, a recent study demonstrated that local mRNA translation is essential for efficient phagocytic activity in microglia, a process central to their ability to promote pain hypersensitivity (31). Despite accumulating evidence of increased expression of translation machinery components following peripheral nerve injury, and the central role of local protein synthesis in microglial phagocytosis, the functional role of microglial protein synthesis in neuropathic pain remains unknown.

In this study, we assessed microglial protein synthesis following peripheral nerve injury using metabolic labeling and manipulated translation in microglia using mouse genetics. We found that peripheral nerve injury in mice resulted in increased rates of protein synthesis in spinal microglia. Furthermore, we demonstrated that increasing translation selectively in microglia was sufficient to trigger their transition to a reactive state and induce mechanical pain hypersensitivity in both sexes, whereas spontaneous pain was increased only in males. Conversely, inhibiting microglial translation decreased nerve injury–induced microglia activation and alleviated neuropathic pain in both sexes. Our findings demonstrate that elevated microglial translation following peripheral nerve injury plays a functional role in promoting pain hypersensitivity.

## Results

### Nascent protein synthesis is increased in microglia after peripheral nerve injury.

**Figure 1:**
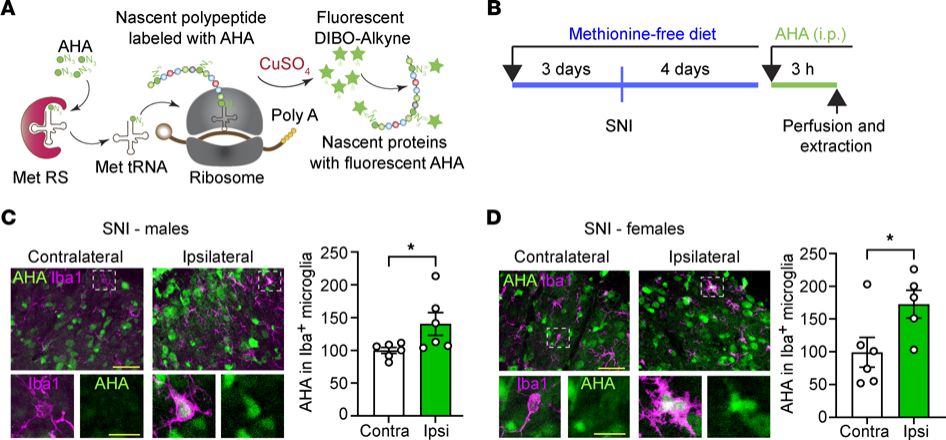
Increase in microglial protein synthesis after peripheral nerve injury. Protein synthesis in spinal microglia was assessed using FUNCAT. (**A**) Schematic illustration of FUNCAT. DIBO; Met RS, methionyl-tRNA synthetase; Met tRNA, methionine transfer RNA. (**B**) Mice were fed a methionine-deficient diet for 3 days before and 4 days after SNI. On day 4 after SNI, AHA was injected, and spinal cords were collected 3 hours later. (**C** and **D**) Immunostaining against Iba1 was used to identify microglia. AHA signal in Iba1^+^ microglia was assessed on the ipsilateral and contralateral sides of injury in males (representative images and quantification normalized to contralateral side in **C**; *n* = 6 mice per group) and females (representative images and quantification normalized to contralateral side in **D**; *n* = 6 mice per group). Unpaired 2-tailed *t* test was used for statistical analyses. Data are plotted as mean ± SEM. *P < 0.05. Scale bar: 30 μm for low-magnification images and 10 μm for high-magnification images.

We first investigated whether peripheral nerve injury induces an upregulation of protein synthesis in spinal microglia. To measure the rates of de novo protein synthesis in microglia, we employed fluorescent noncanonical amino acid tagging (FUNCAT) (32, 33). In FUNCAT, an azide-bearing methionine analog, l-azidohomoalanine (AHA), is incorporated into a newly synthesized polypeptide chain, followed by its labeling in spinal cord sections with click chemistry and detection using immunofluorescence (Figure 1A). Mice were kept on a methionine-deficient diet for 3 days prior to being subjected to the spared nerve injury (SNI) assay of neuropathic pain (34, 35). Four days after SNI, AHA was injected i.p. (Figure 1B), and spinal cord tissue was collected 3 hours later. Quantification of AHA signal in individual Iba1^+^ microglia in the superficial dorsal horn (laminae I, II, and III; identified using a lamina overlay by NeuN staining) showed that 4 days after SNI, microglia in the ipsilateral dorsal horn exhibited an increase in protein synthesis compared with those in the contralateral side in both male and female mice (males in Figure 1C, females in Figure 1D; see also Supplemental Figure 1A; supplemental material available online with this article; https://doi.org/10.1172/JCI180190DS1). An assessment at a later time point, 14 days after SNI, revealed no significant increase in microglial protein synthesis (Supplemental Figure 1B). The validity of the FUNCAT approach for detecting protein synthesis was confirmed using the protein synthesis inhibitor anisomycin, which blocked AHA incorporation (Supplemental Figure 1C). Thus, protein synthesis was upregulated in spinal microglia on day 4 but not on day 14 after peripheral nerve injury.

### Activating 4E-BP1–dependent translation in microglia induces mechanical hypersensitivity.

**Figure 2:**
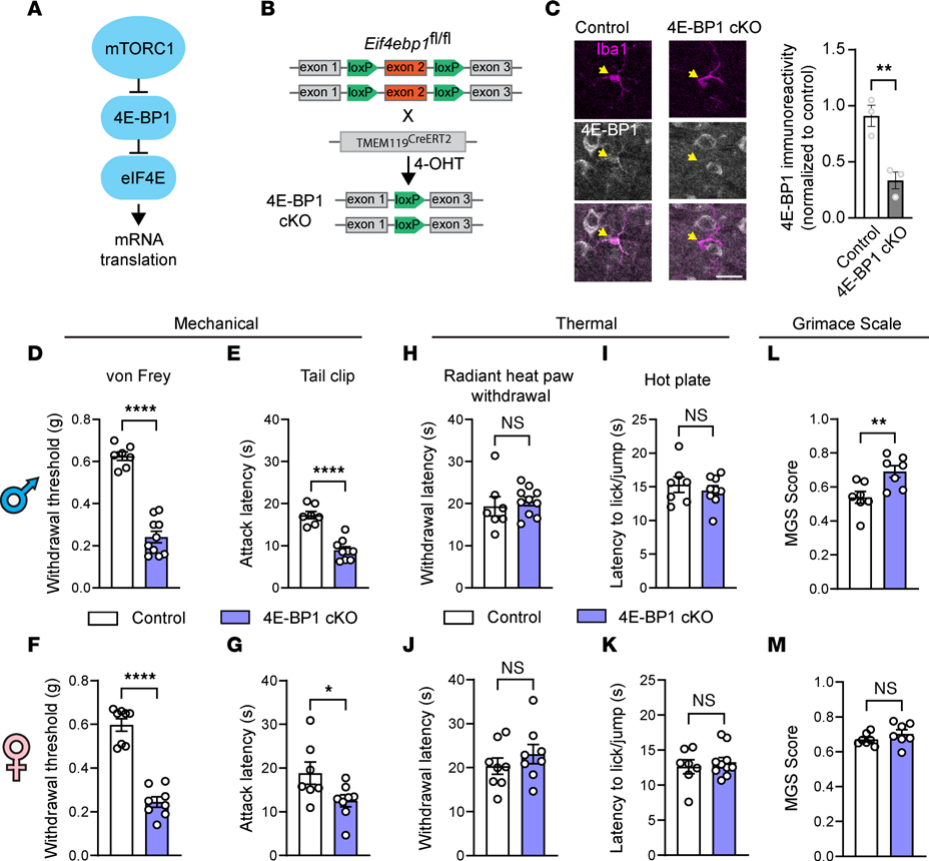
Increase in microglial translation induces mechanical pain hypersensitivity. (**A**) Schematic illustration depicting regulation of mRNA translation via the mTORC1/4E-BP1 axis. (**B**) Generation of mice with conditional ablation of 4E-BP1 in microglia under the *TMEM119^CreERT2^* promoter. (**C**) IHC against 4E-BP1 confirmed reduced levels of 4E-BP1 in 4E-BP1–cKO mice (*n* = 3 mice/group). Scale bar: 20 μm. Yellow arrows mark the location of microglia. Control (*TMEM119^CreERT2^*) and 4E-BP1–cKO mice were tested for mechanical and thermal sensitivity. Mechanical sensitivity was tested using von Frey (males in **D** and females in **F**) and tail-clip (males in **E** and females in **G**) assays. Thermal sensitivity was tested using radiant heat paw-withdrawal (males in **H** and females in **J**) and hot-plate (males in **I** and females in **K**) assays. Spontaneous pain was assessed using the mouse grimace scale (MGS; males in **L** and females in **M**). In behavioral experiments, *n* = 7–10 mice per group. For analyses, unpaired 2-tailed *t* test was used. Two-way ANOVA was used to assess sex differences in the MGS test (**L** and **M**), revealing a significant main effect of sex [*F_(1,24)_* = 6.548, *P* = 0.0172]. Tukey’s multiple-comparison test showed a difference between control and 4E-BP1–cKO males (*P* = 0.0041), but not between females (*P* = 0.8575). Data are plotted as mean ± SEM. *P < 0.05, **P < 0.01, ****P < 0.0001.

To study the functional role of enhanced microglial protein synthesis in pain, we assessed the effect of increasing translation in microglia on pain thresholds. mTOR complex 1 (mTORC1) is a master regulator of mRNA translation. mTORC1 stimulates translation predominantly by phosphorylating eukaryotic initiation factor (eIF) 4E-binding proteins (4E-BPs), which repress translation by binding and inhibiting the activity of the mRNA 5′ cap-binding protein eIF4E (Figure 2A). Phosphorylation of 4E-BPs by mTORC1 induces their dissociation from eIF4E, promoting formation of an eIF4F complex (composed of eIF4E, eIF4A, and eIF4G), which stimulates cap-dependent translation (36, 37). Ablation of 4E-BPs mimics mTORC1-dependent phosphorylation of 4E-BPs and is commonly used to stimulate eIF4E-dependent translation downstream of mTORC1 (38, 39). There are 3 4E-BP isoforms (4E-BP1, 4E-BP2, and 4E-BP3; 4E-BP3 expression levels are very low in the central nervous system), which have similar functions but differ in tissue distribution (40). We previously showed that 4E-BP1 is the main isoform modulating nociception, as whole-body 4E-BP1–KO (but not 4E-BP2–KO) mice exhibited reduced mechanical pain thresholds (40).

To stimulate translation in microglia, we ablated 4E-BP1 selectively in microglia by crossing *Eif4ebp1^fl/fl^* mice with mice expressing Cre recombinase under the microglia-specific *Tmem119* promoter to generate *Eif4ebp1^fl/fl^:TMEM119^CreERT2^* mice (referred to as 4E-BP1–cKO; Figure 2B). To induce Cre-dependent ablation of 4E-BP1 in microglia, we injected 4-hydroxytamoxifen (4-OHT) i.p. on P4–P9. IHC confirmed reduction of 4E-BP1 in Iba1^+^ microglia in the spinal cord of 4E-BP1–cKO mice compared with control (*Eif4ebp1^fl/fl^*) animals (Figure 2C). Behavioral analysis revealed that adult male and female 4E-BP1–cKO mice exhibited mechanical hypersensitivity in von Frey (Figure 2, D and F) and tail-clip (Figure 2, E and G) assays. Consistent with the previous report in whole-body 4E-BP1 knockouts, thermal sensitivity was not altered in 4E-BP1–cKO mice in the radiant heat paw-withdrawal (Figure 2, H and J) and hot-plate (Figure 2, I and K) assays. Assessment of spontaneous pain using the mouse grimace scale (MGS) revealed an increase in the MGS score in 4E-BP1–cKO male mice (Figure 2L) but not in females (Figure 2M).

### Translational upregulation induces morphological changes and enhanced phagocytosis in microglia.

**Figure 3:**
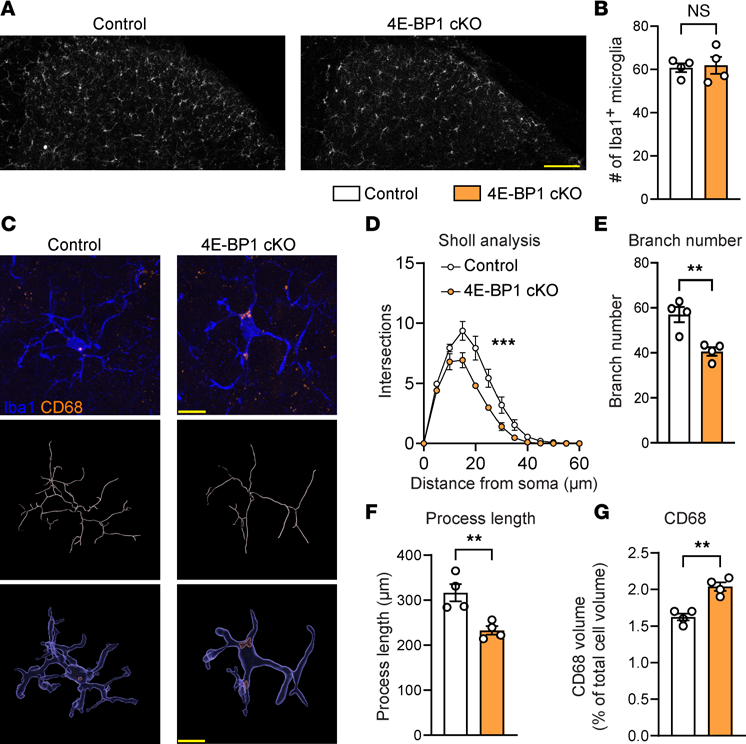
Phenotypic changes in microglia with enhanced translation. (**A**) The number of Iba1^+^ microglia in the dorsal horn is not different in 4E-BP1–cKO mice compared with control (*TMEM119^CreERT2^*) animals (representative images in **A** and quantification in **B**, *n* = 4 mice per group). Scale bar: 100 μm. 3D analysis of Iba1^+^ microglia revealed that 4E-BP1–cKO microglia exhibit changes in Sholl analysis results (**C**: representative images, skeleton and volumetric reconstructions; **D**: quantification; *n* = 4 mice/group), branch number (**E**: *n* = 4 mice /group), process length (**F**: *n* = 4 mice /group), and volumetric analysis of CD68 (**C**: representative 3D volume renders reconstruction images; **G**: quantification, as a percentage of the total cell volume; *n* = 4 mice/group). Scale bar: 10 μm. Unpaired 2-tailed *t* test was used for **B**, **E**, **F**, and **G**. Two-way ANOVA followed by Tukey’s post hoc comparison was used for **D**. Data are plotted as mean ± SEM. **P < 0.01, ***P < 0.001.

We next studied the effect of enhancing protein synthesis in microglia on their number, morphology, and phagocytic capacity. 4E-BP1–cKO mice exhibited no differences in microglial density in the lumbar spinal cord dorsal horn (Figure 3, A and B). Morphological analysis of microglia using 3D reconstruction of Iba1 staining revealed that 4E-BP1–deficient microglia exhibited smaller arbors, indicated by the decreased number of intersections across the arbor in 3D Sholl analysis (Figure 3, C and D) and reduced branch number (Figure 3E) and length (Figure 3F). Quantification of lysosomal volume in microglia using volumetric analysis of CD68 signal demonstrated an increased volume of CD68^+^ lysosomes in Iba1^+^ microglia in 4E-BP1–cKO mice (Figure 3, C and G). The reduced arborization and increased phagocytic capacity observed in 4E-BP1–deficient microglia suggest that stimulation of protein synthesis promoted the transition of microglia to a more reactive state (41).

### 4E-BP1 ablation induces changes in microglial translational landscape.

**Figure 4:**
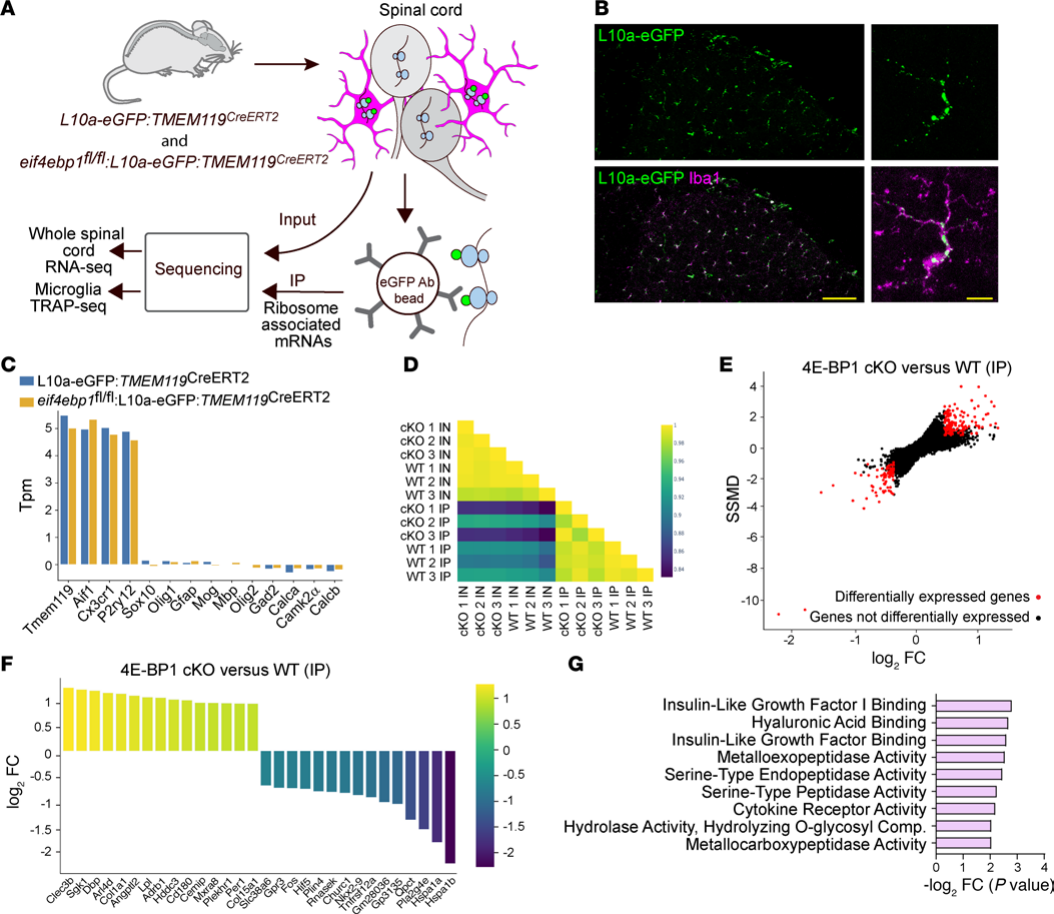
TRAP RNA-Seq reveals differentially expressed genes in 4E-BP1–cKO microglia. (**A**) Schematic illustration of the TRAP approach. (**B**) Imaging of spinal cord section from L10a-eGFP:*TMEM119^CreERT2^* mice confirmed the presence of L10a-eGFP in Iba1^+^ microglia. Scale bars: 100 μm (left) and 10 μm (right). (**C**) Microglial markers are enriched and nonmicroglial markers are depleted in IP fractions. (**D**) Heatmap of the correlation coefficients between different samples (IN, input; IP, immunoprecipitated). (**E**) Dual-flashlight plot shows SSMD versus log_2_ FC for genes in IP samples. Positive log_2_ FC indicates increased expression in 4E-BP1–cKO mice. Parameters for defining data as upregulated or downregulated in 4E-BP1–cKO are: increased, SSMD ≥ 0.9, BC ≥ 0.5, FC ≥ 1.33; decreased, SSMD ≤ 0.9, Bhattacharyya coefficient ≤ 0.5, FC ≤ 1.33. (**F**) Top 15 upregulated and downregulated genes in IP fractions in 4E-BP1–cKO versus control mice. (**G**) Gene Ontology (GO) analysis of the top 100 upregulated genes (Enrichr, GO Molecular Function).

Ablation of 4E-BP1 stimulates translation of eIF4E-sensitive mRNAs, which are involved in immune responses, modulation of extracellular matrix, cell survival, and growth (42–45). To better understand how 4E-BP1 ablation affects microglial functions and induces mechanical pain hypersensitivity, we studied changes in the translational landscape in microglia lacking 4E-BP1. To identify the actively translating mRNAs in microglia, we employed a translating ribosome affinity purification (TRAP) approach (46, 47). In the TRAP assay, the ribosomal subunit L10a tagged with eGFP (L10a-eGFP) is expressed in a specific cell type (Figure 4A). IP with eGFP antibody captures the eGFP-tagged ribosomes, and subsequent sequencing of immunoprecipitated ribosome-bound mRNAs allows for the identification of actively translated mRNAs in the cell type of interest.

To this end, we generated control (L10a-eGFP:*TMEM119^CreERT2^*) and 4E-BP1–cKO (*Eif4ebp1^fl/fl^*:L10a-eGFP:*TMEM119^CreERT2^*) mice expressing L10a-eGFP in microglia. Imaging of spinal cord sections confirmed that L10a-eGFP was expressed in Iba1^+^ microglia (Figure 4B). We then performed IP for eGFP in spinal cord lysates (3 biological replicates per genotype, 3 mice pooled per replicate) and sequenced the IP- and input-associated (IP- and IN-associated) RNAs in both groups. A high correlation of sequencing reads between biological replicates (*r* ≥ 0.97 for IN and for IP, Supplemental Figure 2, A–D) indicated reproducibility across experiments. The enrichment of microglial gene markers (e.g., *Tmem119*, *Aif1*, *Cx3cr1*, *P2ry12*) and depletion of neuronal (e.g., *Gad2*, *Camk2a*, *Calca*, *Calcb*) and other nonneuronal (e.g., *Sox10*, *Olig1*, *Gfap*, *Mog*, *Mbp*, *Olig2*) gene markers in immunoprecipitated mRNA populations (Figure 4C) demonstrated the specificity of the approach. Analysis of ribosome-bound mRNAs in microglia of 4E-BP1–cKO and control mice revealed an increase in ribosome occupancy for 135 genes and a decrease for 79 genes (Figure 4, D–F; a complete dataset is provided in Supplemental Table 1). Normalization of ribosome-bound mRNA reads to total transcript reads from IN fractions (IP/IN ratio, Supplemental Figure 2, E and F) provided a proxy for translational changes. Pathway analysis of genes with altered ribosome occupancy in 4E-BP1–cKO mice showed changes in processes related to the insulin-like growth factor 1 (IGF1) pathway, extracellular matrix organization (hyaluronic acid binding, metalloexopeptidase and hydrolase activity, serine-type peptidase activity), and cytokine receptors (Figure 4G), which are implicated in regulating microglial functions (48) and pathological pain (14, 49, 50). Additionally, several mRNAs encoding proteins involved in phagocytosis (*Itgb3*, *AXL*, *Cd44*, and *Cdc42*) exhibited increased ribosome binding in 4E-BP1–KO microglia (Supplemental Table 1). In response to peripheral nerve injury, spinal microglia promote pain hypersensitivity by phagocytosing various cellular components, including inhibitory synapses (12, 13) and extracellular matrix components (14). At later stages, a subpopulation of CD11c^+^ microglia facilitates pain resolution by engulfing myelin debris (e.g., myelin basic protein [MBP]) and secreting IGF1 (50, 51). Assessment of microglial lysosomal content revealed an increased proportion of microglia containing VGAT (vesicular GABA transporter), a marker of inhibitory synapses, and WFA (*Wisteria floribunda* agglutinin), a marker of the extracellular matrix — but not of MBP — within CD68^+^ lysosomes of 4E-BP1–cKO mice (Supplemental Figure 3, A–C). scRNA-Seq of sorted microglia (gated for CD45^lo^CD11B^hi^CX3CR1^hi^) did not reveal substantial changes in the proportions of cells in distinct microglial subpopulations or the appearance of new microglial clusters in 4E-BP1–cKO mice (Supplemental Figure 4, A and B). In 4E-BP1–cKO mice, distinct microglial clusters (clusters 4 and 5) showed similarities to the transcriptional signature of disease-associated microglia (DAM) and injury-responsive microglia (IRM) (52, 53), but not of CD11c^+^ microglia (50) (Supplemental Figure 4, C–E). Administration of the IGF1-neutralizing antibody (50) did not alleviate mechanical hypersensitivity in 4E-BP1–cKO mice (Supplemental Figure 4F), suggesting that the increased pain hypersensitivity in these mice was not mediated through CD11c^+^ microglia/IGF1 signaling.

### Inhibiting translation in microglia alleviates mechanical hypersensitivity in neuropathic pain.

**Figure 5:**
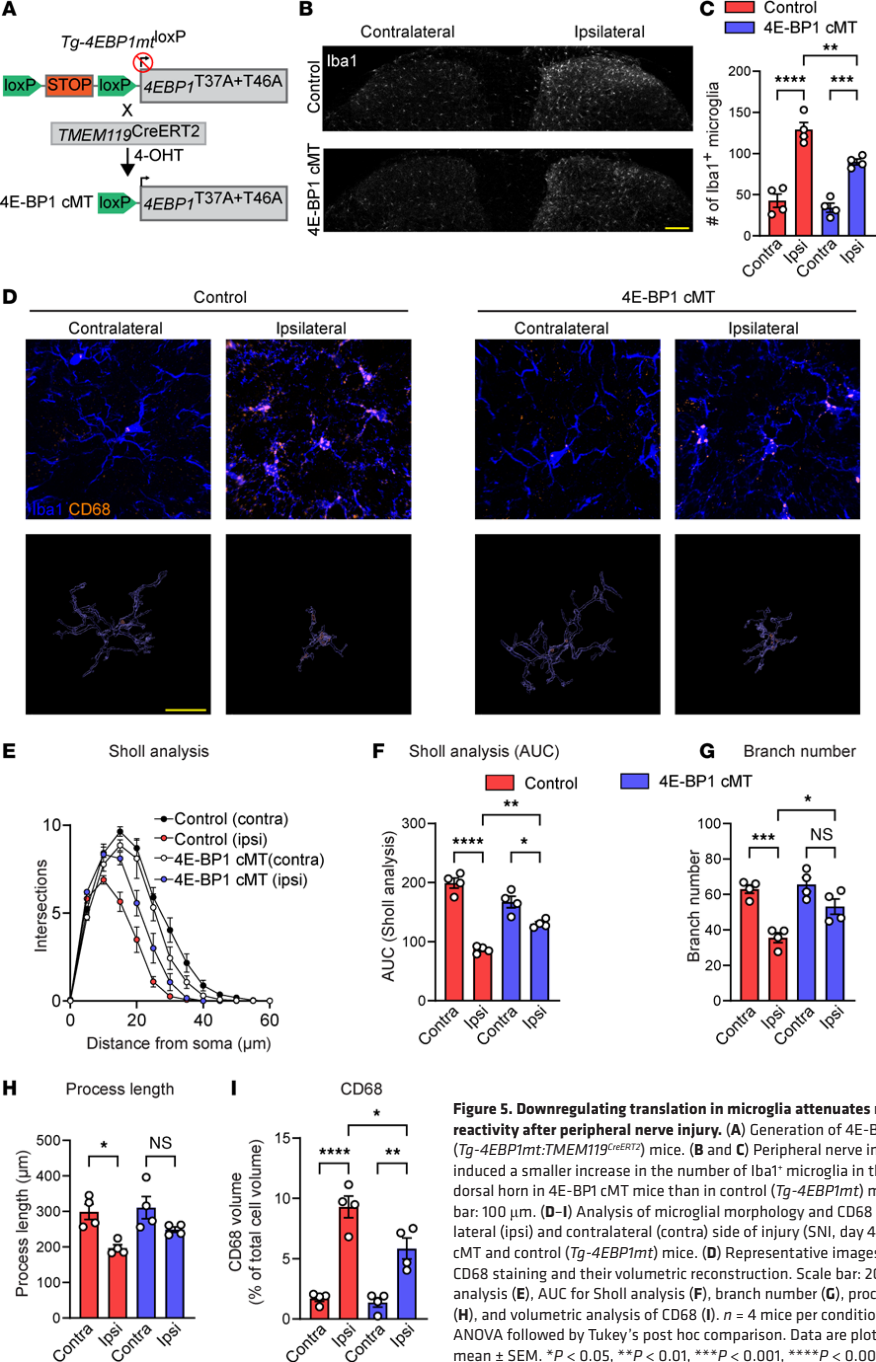
Downregulating translation in microglia attenuates microglial reactivity after peripheral nerve injury. (**A**) Generation of 4E-BP1 cMT (*Tg-4EBP1mt:TMEM119^CreERT2^*) mice. (**B** and **C**) Peripheral nerve injury (SNI) induced a smaller increase in the number of Iba1^+^ microglia in the lumbar dorsal horn in 4E-BP1 cMT mice than in control (*Tg-4EBP1mt*) mice. Scale bar: 100 μm. (**D**–**I**) Analysis of microglial morphology and CD68 on the ipsilateral (ipsi) and contralateral (contra) side of injury (SNI, day 4) in 4E-BP1 cMT and control (*Tg-4EBP1mt*) mice. (**D**) Representative images of Iba1 and CD68 staining and their volumetric reconstruction. Scale bar: 20 μm. Sholl analysis (**E**), AUC for Sholl analysis (**F**), branch number (**G**), process length (**H**), and volumetric analysis of CD68 (**I**). *n* = 4 mice per condition. Two-way ANOVA followed by Tukey’s post hoc comparison. Data are plotted as mean ± SEM. *P < 0.05, **P < 0.01, ***P < 0.001, ****P < 0.0001.

We next studied whether inhibiting translation in microglia alleviates nerve injury–induced microglial reactivity and mechanical pain hypersensitivity. To selectively inhibit translation in microglia, we used a transgenic mouse conditionally expressing 4E-BP1 with mutations in 2 key mTORC1 phosphorylation sites (threonine to alanine at amino acid positions 37 and 46; *Tg-4EBP1mt*) (54). Crossing *Tg-4EBP1mt* mice to *TMEM119^CreERT2^* animals (*Tg-4EBP1mt:TMEM119^CreERT2^*, referred to as 4E-BP1 cMT; Figure 5A) allows for the expression of mutated 4E-BP1 in microglia, resulting in the diminished effect of mTORC1 on eIF4E-dependent translation in microglia (54).

**Figure 6:**
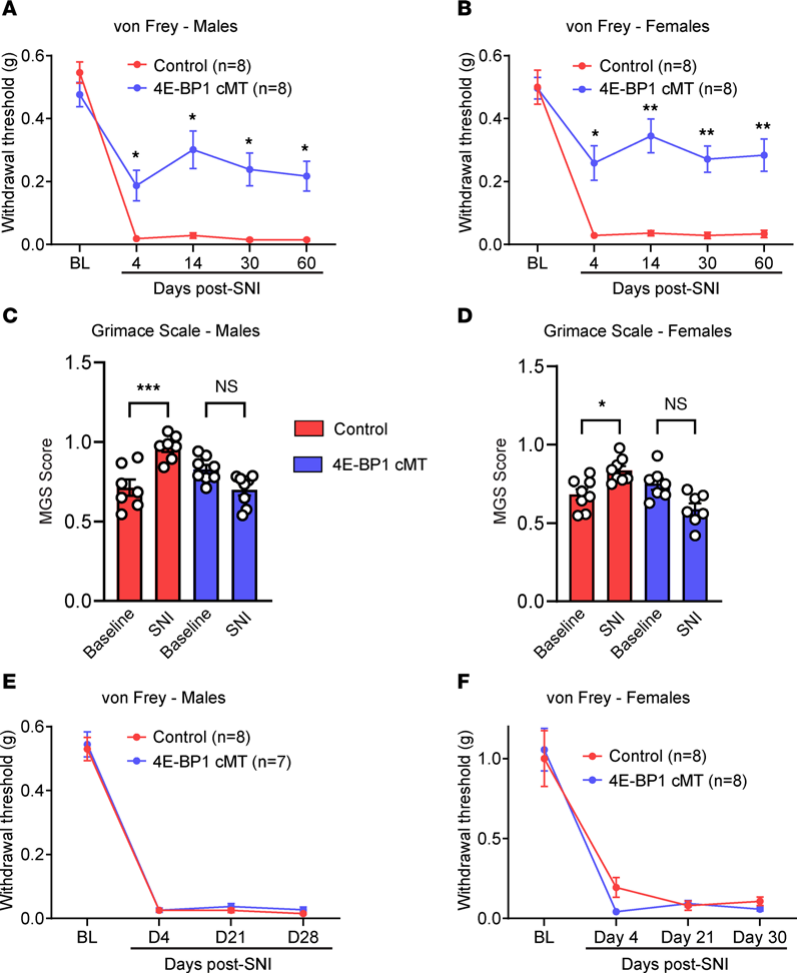
Downregulating translation in microglia alleviates neuropathic pain. Paw-withdrawal thresholds (von Frey assay) were tested in 4E-BP1 cMT and control (*Tg-4EBP1mt*) male (**A**) and female (**B**) mice after SNI (*n* = 8 mice per group). BL, baseline. MGS was used to assess spontaneous pain on day 4 after SNI in male (**C**) and female (**D**) 4E-BP1 cMT and control mice (*n* = 7–8 mice per group). Mutant 4E-BP1 expression was induced in microglia after pain hypersensitivity was established by peripheral nerve injury via injection of tamoxifen to 4E-BP1 cMT (and control, *Tg-4EBP1mt*) male (**E**) and female (**F**) mice from day 4 to 9 after SNI. Two-way ANOVA followed by Tukey’s post hoc comparison was used for **A**, **B**, **E**, and **F**. One-way ANOVA followed by Tukey’s post hoc comparison was used for **C** and **D**. Data are plotted as mean ± SEM. *P < 0.05, **P < 0.01, ***P < 0.001.

Peripheral nerve injury (SNI) in control *Tg-4EBP1mt* animals induced an increase in the number of Iba1^+^ microglia on the ipsilateral side on day 4 after SNI (Figure 5, B and C). In 4E-BP1 cMT mice, SNI also induced microgliosis; however, the increase in the number of microglia was smaller than in the control group. Furthermore, nerve injury–induced phenotypic switch of microglia to a reactive state, as shown by decreased arborization (Figure 5D; Sholl analysis in Figure 5, E and F; branch number in Figure 5G; process length in Figure 5H) and increased volume of CD68^+^ lysosomes (Figure 5, D and I) were attenuated in 4E-BP1 cMT mice. Behavioral analysis revealed no change in baseline mechanical sensitivity in 4E-BP1 cMT mice (Figure 6, A and B). However, mechanical hypersensitivity induced by peripheral nerve injury was substantially alleviated in 4E-BP1 cMT compared with control animals, in both male (Figure 6A) and female (Figure 6B) mice. Furthermore, while peripheral nerve injury caused an increase in the MGS score in control animals on day 4 after SNI, the increase was not observed in 4E-BP1 cMT male and female mice (Figure 6, C and D). Finally, we tested the effect of reducing eIF4E-dependent translation in microglia after peripheral nerve injury, when hypersensitivity is fully developed. Inducing mutant 4E-BP1 expression in microglia after SNI (by administering tamoxifen to 4E-BP1 cMT mice from days 4 to 9 after SNI) did not alleviate nerve injury–induced mechanical hypersensitivity (Figure 6, E and F), suggesting that increased 4E-BP1–dependent microglial translation plays a role in the development, but not the maintenance, of neuropathic pain.

## Discussion

In this work, we found that peripheral nerve injury enhanced protein synthesis in spinal microglia. Furthermore, we demonstrated the functional role of increased protein synthesis, as upregulating 4E-BP1–dependent translation in microglia induced the transition of microglia to a reactive state and promoted pain hypersensitivity, whereas translational downregulation attenuated nerve injury–induced microglia activation and pain.

Transcriptional profiling is commonly used to investigate molecular processes and define cellular states; however, accumulating evidence indicates that the regulation of gene expression at the level of mRNA translation has a major role in determining the cellular proteome and regulating diverse cellular functions. Translational regulation allows for rapid and local modulation of protein levels in response to internal and external cues (55). The central role of local translation in mediating efficient microglial phagocytosis has recently been demonstrated (31). This work, along with other studies (56–60), underscore the importance of translational control in microglia in regulating key phenotypes and functions. Our findings bolster this conclusion by showing that stimulation of 4E-BP1–dependent translation in microglia was sufficient to induce phenotypes associated with microglial activation (reduced arborization and increased lysosome volume), and inhibition of translation attenuated the transition of microglia from a resting to a reactive state. These findings have substantial implications for understanding the mechanisms modulating microglial activity in the context of neuropathic pain. They may also be relevant for other conditions where mTORC1 activity is enhanced, such as germline or somatic mutations in *PTEN*, *TSC1/2*, *PIK3CA*, *AKT3*, *RHEB*, and *MTOR* (61–68). Collectively, these results deepen our understanding of the intricate mechanisms that promote microglial reactivity and their roles in regulating neuronal circuits and brain functions.

The increase in microglial protein synthesis was detected on day 4 after nerve injury but not on day 14. Moreover, downregulating 4E-BP1–dependent translation before peripheral nerve injury, but not at later stages (injection of tamoxifen on days 4–9 after SNI in 4E-BP1 cMT mice), alleviated pain hypersensitivity. These results indicate that the nerve injury–induced enhancement of microglial protein synthesis was involved in the development, but not maintenance, phase of neuropathic pain. They also suggest that the demonstrated role of microglia in neuropathic pain maintenance (69, 70) is not mediated via a 4E-BP1–dependent translational control mechanism.

Microglia can promote development of pain hypersensitivity via numerous mechanisms, including release of bioactive molecules (e.g., cytokines and chemokines) and phagocytosis of synaptic elements and components of the extracellular matrix. The microglial TRAP analysis identified increased ribosome binding of several mRNAs encoding proteins involved in phagocytosis, such as *Itgb3*, *AXL*, *Cd44*, and *Cdc42*, in 4E-BP1–cKO mice. Furthermore, 4E-BP1–cKO mice had more microglia containing lysosomal VGAT (a marker of inhibitory presynaptic terminals preferentially engulfed by microglia in SNI) (12) and WFA (a marker of extracellular matrix engulfed by microglia in SNI) (14), raising the possibility that pain hypersensitivity in 4E-BP1–cKO mice was caused, at least in part, by increased phagocytosis of inhibitory synapses and components of the extracellular matrix. Distinct microglia subpopulations may have pronociceptive or antinociceptive functions. CD11c^+^ antinociceptive microglia promote the resolution of neuropathic pain via IGF1-dependent mechanism (50). Because the quantity of CD11c^+^ microglia is very low in naive animals, a further decrease in their abundance in 4E-BP1–cKO mice is unlikely. Accordingly, in 4E-BP1–cKO mice, we did not find microglia harboring a CD11c-like transcriptome, and IGF1 inhibition had no effect on mechanical hypersensitivity, suggesting that CD11c^+^ microglia and IGF1 signaling do not substantially contribute to pain phenotypes in 4E-BP1 cKOs.

We found that activation of microglial translation induced mechanical but not thermal hypersensitivity. This finding is consistent with our previous report that whole-body 4E-BP1–KO mice exhibit differential mechanical but not thermal hypersensitivity compared with WT. Previous work demonstrated that microglia play a role in promoting both mechanical and thermal hypersensitivity following tissue injury (71, 72), suggesting that the modality specificity in 4E-BP1–cKO mice is related to the effect of 4E-BP1. 4E-BP1 regulates translation of a subset of mRNAs that are eIF4E sensitive (42–45). These mRNAs have a long and highly structured 5′ UTR that requires the eIF4F complex and its eIF4A helicase activity to unwind mRNA secondary structures to promote translation. Using TRAP, we identified transcripts showing altered ribosome occupancy in 4E-BP1–deficient microglia. Pathway analysis of the identified transcripts revealed changes in the IGF-1, extracellular matrix organization, and cytokine receptor pathways, which are involved in regulation of microglia functions and pain (14, 48–50). Additionally, several transcripts encoding proteins involved in phagocytosis (*Itgb3*, *Axl*, *Cd44*, and *Cdc42*) showed increased ribosome binding in the TRAP analysis of 4E-BP1–KO microglia, potentially linking 4E-BP–dependent translation and enhanced phagocytosis. Why altered translation of these transcripts promotes mechanical but not thermal hypersensitivity remains unknown.

We found that increasing translation in microglia (using 4E-BP1 cKO) was sufficient to induce mechanical hypersensitivity in both male and female mice. It also induced spontaneous pain, as assessed using the MGS; however, this phenotype was observed in males only. Inhibiting translation in microglia alleviated nerve injury–induced pain hypersensitivity in both sexes. Although many studies have demonstrated that microglia are required for the development of neuropathic pain exclusively in males (15), emerging evidence suggests that not all microglial functions in pain are sexually dimorphic and that the mode and timing of microglial modulation or inhibition, as well as the pain model employed, dictate the role of microglia in females (72–74). Induction of spontaneous pain in male but not female mice with increased microglial translation indicates sexual dimorphism in this behavior. The mechanisms and signaling pathways stimulating translation in spinal microglia following peripheral nerve injury remain unknown. Activation of microglial MAPKs, such as p38 and ERK, or mTORC1, which play a central role in neuropathic pain and promote translation by modulating eIF4E activity (23–28, 75, 76), might be involved.

Notably, peripheral nerve injury also enhances protein synthesis in distinct neuronal cell types in the dorsal horn of the spinal cord, predominantly in inhibitory neurons, which exhibit more prominent translational upregulation in the chronic phases of neuropathic pain (77).

In summary, the demonstration of translational upregulation in microglia following peripheral nerve injury and its contribution to the development of mechanical hypersensitivity enhance our understanding of basic microglial biology and the mechanisms by which microglia promote pathological pain.

## Methods

### Sex as a biological variable

Male and female mice were used for FUNCAT (Figure 1 and Supplemental Figure 1B) and behavioral experiments (Figures 2 and 6); all other experiments were performed on male mice.

### Animals

*Eif4ebp1^fl/fl^* mice (78) and *Tg-4EBP1mt* mice (54) were crossed to mice that express Cre recombinase under the microglia-specific promotor *TMEM119^CreERT2^* (The Jackson Laboratory, JAX stock 031820) (79) to generate *Eif4ebp1^fl/fl^:TMEM119^CreERT2^* conditional KO and *Tg-4EBP1mt:TMEM119^CreERT2^* mutant mice, respectively. *Eif4ebp1^fl/fl^*:L10a-eGFP:*TMEM119^CreERT2^* and L10a-eGFP:*TMEM119^CreERT2^* mice (L10a-eGFP; ref. 47) were generated for TRAP-Seq. At 3 weeks of age, mice were weaned, and ear notch samples were collected for genotyping using PCR. *Eif4ebp1*-transgenic and WT alleles were detected using PCR assay in which primer *Eif4ebp1* F (5′-CACATTTCAGGGAGAGGGTGATG-3′) and primer *Eif4ebp1* R (5′-GCTGGGTTCTAAGAGTGGTACTTT-3′) amplified a 250 bp fragment (WT) and a 347 bp fragment (exon 2 of the *Eif4ebp1* conditional allele). Cre expression was detected by PCR with primers Cre F (5′-GATCTCCGGTATTGAAACTC-3′) and Cre R (5′-GCTAAACATGCTTCATCGTC-3′), which amplify a 650 bp fragment. All experiments were performed on 8- to10-week-old mice, and the experimenter was masked to the genotype/condition in all studies. All procedures complied with Canadian Council on Animal Care guidelines and were approved by the McGill University Downtown Animal Care Committee.

### 4-OHT and tamoxifen

4-OHT was resuspended at 20 mg/mL in ethanol and diluted in sunflower seed oil at a final concentration of 2 mg/mL in 10% ethanol. Mice were administered 4-OHT (MilliporeSigma, H7904) i.p., once a day, from P4 to P9 for a total dosage of 0.25 mg per animal.

For induction of Cre expression after SNI, mice were administered tamoxifen. Tamoxifen (MilliporeSigma, T5648) was prepared in sunflower seed oil at a stock concentration of 10 mg/mL. Starting 4 days after SNI, tamoxifen (100 mg/kg body weight) was administered i.p. once a day for 5 consecutive days.

### SNI

Neuropathic pain was induced using the SNI model (34, 35). Mice were anesthetized with 2% isoflurane. A small incision was made to the upper thigh. The sciatic nerve was exposed after cutting through the femoris muscle. A clamp was used to keep the incision open, while ligating (7.0 silk Covidien, S-1768K) the tibial and common peroneal branches of the sciatic nerve, leaving the sural nerve intact. After ligation, a 2–4 mm section of the 2 branches was removed. The muscle and skin were closed and sutured (Vicryl, J489G, Ethicon).

### FUNCAT

Male and female mice at 8–10 weeks of age were fed a methionine-deficient diet for 1 week (TD.110208, Envigo RMS Inc.) and were weighed daily to ensure weight loss did not exceed 20% of initial weight. After 7 days, mice received an i.p. injection of AHA (100 μg/g body weight, i.p.; Click-IT AHA, C10102, Thermo Fisher Scientific). Mice were anesthetized and intracardially perfused 3 hours after injection with PBS, followed by 4% paraformaldehyde (PFA) in 0.1 M phosphate buffer, pH 7.4. Spinal cord tissue was extracted and kept in 4% PFA overnight, before being transferred to PBS. Tissue was transversely cut into 30 μm sections, washed, and blocked overnight in a blocking solution (10% normal goat serum, 0.5% Triton-X100, and 5% sucrose in PBS). The following day, click chemistry (200 μM triazole ligand, 400 μM TCEP, 2 μM fluorescent Alexa Fluor 555 alkyne [A20013, Thermo Fisher Scientific], and 200 μM CuSO_4_ in PBS) was performed on the spinal cord sections, followed by overnight incubation at 4°C. IHC was then performed (described below) on sections. Images were taken using a Zeiss confocal microscope (LSM 880) with 63×/1.40 Oil DIC f/ELYRA objective. Integrated density of FUNCAT signal within Iba^+^ cells in the superficial dorsal horn (laminae I, II, and III, defined using a lamina overlay based on NeuN staining) was quantified using ImageJ (NIH) on maximum-intensity projection images. Background noise was subtracted from final calculations. Three images were taken from each section of the ipsilateral side and the contralateral side of injury (*n* = 5–6 mice/group). FUNCAT signal within all microglia in the image (~5–10 cells per image) were quantified. The values from the total microglia were averaged to obtain a single value per mouse.

### IHC

Mice were perfused intracardially with PBS, followed by 4% PFA in 0.1 M phosphate buffer, pH 7.4. For 3D image analysis, tissue was transversely cut into 50 μm sections. All other imaging experiments were done on transversely cut 30 μm sections. Sections were washed 3 times with Triton-X100 in PBS (PBS-T) at 10-minute increments and blocked for 2 hours at room temperature in a solution of 10% normal goat serum (NGS) and 0.2% PBS-T, before overnight incubation with primary antibodies. For 3D image analysis, Iba1 (1:500, 019-19741, Wako) and CD68 (1:500, MCA1957, Bio-Rad) in 0.5% PBS-T were used. For confirmation of 4E-BP1 ablation, 4E-BP1 (1:200, 2855S, Cell Signaling Technology) and Iba1 (1:500, 234-308, Synaptic Systems) were used. For FUNCAT, Iba1 (1:500, 234-308, Synaptic Systems) and NeuN (1:1,000, MAB377, Millipore) were used.

The following day, the tissue was washed 3 times at 10-minute intervals in PBS-T and incubated for 2 hours with the corresponding secondary antibody. For 3D image analysis, we used goat anti-rabbit Alexa Fluor 647 (1:500, A-21245, Thermo Fisher Scientific), and donkey anti-rat Alexa Fluor 594 (1:500, A-21209, Thermo Fisher Scientific). For FUNCAT analysis, Alexa Fluor 555 alkyne (A-20013, Thermo Fisher Scientific), goat anti–guinea pig Alexa Fluor 647 (A-21450, Thermo Fisher Scientific), and goat anti-mouse Alexa Fluor 488 (A-21042, Thermo Fisher Scientific) were used. For 4E-BP1 cKO, goat anti–guinea pig Alexa Fluor 647 (A-21450, Thermo Fisher Scientific), goat anti-mouse Alexa Fluor 488 (A-21042, Thermo Fisher Scientific), and goat anti-rabbit Alexa Fluor 568 (A-11036, Thermo Fisher Scientific) were used. After incubation, 3 additional washes were performed in PBS, followed by mounting using Aqua Poly/Mount (Polysciences Inc.).

#### Staining for WFA, VGAT, and MBP.

Triton-X was not used for any staining involving WFA. For microglial engulfment staining, Iba-1 ([1:500], Wako 019-19741), *Wisteria floribunda* agglutinin ([1:100], L1516, MilliporeSigma) and CD68 ([1:500], MCA1957, Bio-Rad) were used. For inhibitory synaptic markers, VGAT ([1:1,000], 131011, Synaptic Systems) was used. As a myelin marker, MBP ([1:1,000], 836504, BioLegend) was used. Secondary antibodies were goat anti-rabbit Alexa Fluor 647 (1:500, A-21245, Thermo Fisher Scientific), donkey anti-rat Alexa Fluor 594 (1:500, A-21209, Thermo Fisher Scientific), goat anti-mouse Alexa Fluor 488 (A-21042, Thermo Fisher Scientific), and Streptavidin Alexa Fluor 488 (S11223, Thermo Fisher Scientific) were applied, and tissue was mounted using ProLong Gold Antifade reagent (P36934, Thermo Fisher Scientific). Each group consisted of a total of *n* = 4 male mice, with 3 sections of spinal cord dorsal horn from each mouse (15 cells per section) being imaged using a Zeiss confocal microscope (LSM 880) with 63×/1.40 Oil DIC f/ELYRA objective. *Z*-stacks were taken at 63× magnification and processed as maximum-intensity projections of confocal *Z*-stacks using ImageJ software (v.1.52). Values were calculated by raw counting of combinations of CD68/WFA/Iba-1, CD68/VGAT/Iba-1, or CD68/MBP/Iba-1 signal compared with control.

### Imaris

Sholl and CD68 lysosome analyses of microglia in the spinal cord were performed using IMARIS (9.7.2) (Oxford Instruments, RRID:SCR_007370). Iba1 was stained by performing IHC and used for microglia analysis, and CD68 was used to analyze lysosomal volume. Spinal cord sections were cut at 50 μm thickness. High-resolution *Z*-stack (40–45 μm *Z*-stacks; *xy* resolution: 0.10 μm; *z*-step 0.2 μm, 1,024 × 1,024 pixels) images were taken using a 63×/1.40 Oil DIC f/ELYRA objective on a Zeiss LSM880 confocal microscope.

Imaris 9.7.1 file converter was used to convert the file type from a CZI file to an IMARIS format. The Filament Tracer module of IMARIS was used to reconstruct microglia for Sholl analysis by selecting the Iba1 channel. After reconstruction, the “filament number of Sholl intersections” data were produced by IMARIS and compiled in an Excel spreadsheet before analysis.

Lysosomal volume was measured by 3D reconstruction and colocalizing CD68 signal inside microglia. The surface rendering model was used to identify microglia and CD68 surfaces to perform volume-based analysis. Surfaces were reconstructed for CD68 followed by Iba1. The CD68 volume that occupied Iba1 was analyzed by IMARIS and compiled in an Excel spreadsheet before analysis. Three sections were imaged and analyzed per mouse. A total of 10–15 cells per section were analyzed. Each 4E-BP1–cKO and 4E-BP1 cMT group consisted of 4 mice (*n* = 4).

### IGF-1–neutralizing antibody

Anti–IGF-1 antibody (AF791-SP) and normal goat IgG (for control) (AB-108-C) were purchased from R&D Systems. Antibodies were intrathecally injected (50 μg/mL in PBS, 5 μL/mouse) in 4E-BP1 TMEM119-cKO and TMEM119 Cre mice (control) for 3 consecutive days. Behavior was assessed 7 days after the first injection.

### Behavior

#### Von Frey assay.

The experimenters were masked to the genotype and conditions for all behavioral tests. Male and female mice were habituated and tested in transparent Plexiglas cubicles (5 × 8.5 × 6 cm) that were placed on a perforated steel floor. For testing, Nylon monofilaments were applied perpendicular to the lateral surface of the hind paw for 3 seconds. The up-and-down method of Dixon was applied to estimate the 50% paw-withdrawal threshold (80).

#### Radiant heat paw-withdrawal assay.

Male and female mice were habituated and tested in transparent Plexiglas cubicles (5 × 8.5 × 6 cm) that were placed on a transparent glass floor. A focused beam of light, set at 20% of the maximum intensity, was applied to the outer surface of the hind paw using IITC Life Science model 390. Latency to paw withdrawal was measured, with a cutoff response time of 40 seconds.

#### Tail-clip test.

Male and female mice were placed in an enclosure (15 × 25 cm). A clip exerting a force of 155*g* was applied 1 cm from the base of the tail. The latency for the mouse to attack the clip was measured. The cutoff for a response was 60 seconds.

#### Hot-plate test.

Male and female mice were placed on a 53°C hot plate apparatus (Columbus Instruments) enclosed in a Plexiglas cylindrical tube. Latency to nociceptive behaviors (jumping, hind paw licking, and rapid shaking of the hind paw) was recorded as a positive response. The cutoff for a positive response was 60 seconds.

#### MGS.

The MGS was implemented as in previous studies (77, 81). Mice were acclimated to custom-made Plexiglas cubicles (5.3 × 8.5 × 3.6 cm) for 1 hour. Following habituation, the mice were recorded for 1 hour using a Sony Digital Camcorder HDR-PJ430V. One frame was selected from each 3-minute interval, resulting in a total of 20 images per mouse. The images were then randomized, and the coder, masked to the experimental groups, analyzed them to calculate each mouse’s mean score. Scoring was based on 5 facial features (action units): orbital tightening, nose bulge, cheek bulge, ear position, and whisker changes. Each action unit was scored on a scale from 0 to 2, where 0 indicated no evidence of the action unit, 1 indicated moderate evidence, and 2 indicated obvious evidence. The final MGS score for each mouse was obtained by averaging the intensity ratings of all 5 features.

### TRAP

TRAP-Seq was performed as previously described (46, 47). The lumbar part of the spinal cord was extracted and transferred to ice-cold dissection buffer (1× HBSS, 2.5 mM HEPES-NaOH [pH 7.4], 35 mM glucose, 5 mM MgCl_2_; 100 μg/mL cycloheximide and 0.2 mg/mL emetine were added just before use). Spinal cords were homogenized in lysis buffer (20 mM HEPES-NaOH [pH 7.4], 12 mM MgCl_2_, and 150 mM KCl in RNAse-free water; 0.5 mM DTT, 1 μl/mL Protease Inhibitor Cocktail [Roche], 100 μg/mL cycloheximide, 20 μg/mL emetine, 40 U/mL RNasin [Promega], and 2 U/mL TURBO DNase [Invitrogen] were added immediately before use) using a glass pestle. The homogenate was then transferred to tubes and further homogenized using a Minilys Personal High Power Tissue Homogenizer (Bertin Technologies) on medium speed for 10 seconds a total of 8 times at 4ºC. Postnuclear samples were collected by centrifuging at 2,000*g* for 5 minutes at 4ºC and collection of the supernatant. NP-40 (AG Scientific) and DHPC (Avanti Polar Lipids) were added to the supernatant at a final concentration of 1% and left to incubate for 5 minutes. A postmitochondrial fraction was obtained by centrifuging samples at 15,000*g* for 10 minutes. An aliquot of 200 μL was taken and used for the IN sample, and the rest of the fraction was incubated in G-coated Dynabeads (10003D, Invitrogen) bound to 50 μg anti-GFP antibodies (HtzGFP-19F7 and HtzGFP-19C8 antibodies were acquired from Memorial Sloan Kettering Center) on an end-over-end mixer overnight. The next day, the beads were washed with a high-salt buffer (containing 20 mM HEPES-NaOH [pH 7.4], 12 mM MgCl_2_, and 0.35 M KCl, 1% NP-40 [AG Scientific] in RNAse-free water; 100 μg/mL cycloheximide and 0.5 mM DTT were added just before use) to collect the IP fraction. RNA was then extracted by incubating the samples in TRIzol (300 μL IP; 600 μL IN) at room temperature for 10 minutes. IP samples were then vortexed and placed on a magnetic stand to remove the extracted RNA from the beads. Equal parts of 100% ethanol were added to each sample (300 μL IP, 800 μL IN). RNA was eluted using a Direct-zol RNA kit (Zymo Research) using the manufacturer’s protocol. The NanoDrop Spectrophotometer ND-1000 (NanoDrop Technologies) was used to quantify RNA yield, and the 2100 Bioanalyzer (Agilent Technologies) was used to assess RNA quality.

### Library generation and sequencing

Sequencing was done on IP and their corresponding input fractions. Spinal cord tissues from male mice, aged 8–10 weeks, were extracted. Control mice and 4E-BP1–cKO mice had 3 replicates; each replicate was obtained from pooling 3 spinal cords.

Total RNA was quantified using a NanoDrop Spectrophotometer ND-1000, and its integrity was assessed on a 2100 Bioanalyzer. rRNA was depleted from 70 ng of total RNA using QIAseq FastSelect (Human/Mouse/Rat 96rxns; QIAGEN). cDNA synthesis was achieved with the NEBNext RNA First Strand Synthesis and NEBNext Ultra Directional RNA Second Strand Synthesis Modules (New England Biolabs). The remaining steps of library preparation were done using the NEBNext Ultra II DNA Library Prep Kit for Illumina (New England Biolabs). Adapters and PCR primers were purchased from New England Biolabs. Libraries were quantified using the KAPA Library Quantification Kits–Complete kit (Universal) (Kapa Biosystems). Average fragment size was determined using a LabChip GX (PerkinElmer) instrument.

The libraries were normalized, pooled, and then denatured in 0.05N NaOH and neutralized using HT1 buffer. The pool was loaded at 175 pM on an Illumina NovaSeq S4 lane using the Xp protocol as per the manufacturer’s recommendations. The run was performed for 2 × 100 cycles (paired-end mode). A PhiX library was used as a control and mixed with libraries at the 1% level. Base calling was performed with RTA v3.4.4. Program bcl2fastq2 v2.20 was then used to demultiplex samples and generate FASTQ reads.

### Bioinformatics and statistical analysis

#### Mapping and TPM quantification.

Raw data quality metrics were examined with FastQC (Bioinformatics, https://www.bioinformatics.babraham.ac.uk/projects/fastqc/), including sequencing quality per base position, adapter content, and duplication levels. Reads were soft-clipped (12 bases per read) to ignore adapters and low-quality bases during alignment using STAR (82) v2.7.6 with the GRCm39 mouse reference genome (Gencode release M31, primary assembly). Reads were also sorted with STAR and then deduplicated with sambamba v0.8.2 (83) prior to quantification with StringTie v2.2.1 (84), which calculated expression levels in transcripts per million (TPM) of all genes for every sample.

#### Order statistics and renormalization of expression data.

Downstream analysis of TRAP datasets was performed as previously described (85). Each transcriptome sample (IN) had consistently expressed genes identified by calculating percentile ranks for each coding gene. We identified 12,783 genes that were above the 30th percentile in each IN sample. Quantile normalization was performed based on the set of all coding genes.

IP (translatome) analysis was performed on the 12,783 consistently transcriptome-expressed genes. To identify consistently expressed genes in the translatome samples, the percentile ranks of TPM were calculated for each of the 12,783 consistently transcriptome-expressed coding genes of each sample. We identified 11,724 of 12,783 genes that were consistently detected in the translatome based on whether their expression was at or above the 15th percentile (out of 12,783 genes) in each IP sample.

#### Differential expression analysis.

Differential expression (DE) analysis was performed as previously described (82). log_2_ fold change (FC) was calculated based on median TPM values for each transcriptome-expressed and translatome-expressed coding gene in the INPUT and IP samples, respectively. Strictly standardized mean difference (SSMD) was used to reveal genes with systematically altered expression percentile ranks between sham-treated and SNI mice of the same cell type and time point. SSMD is the difference of means controlled by the variance of the sample measurements. SSMD measured the effect size to control for within-group variability. Differentially expressed genes were determined between sham-treated and SNI of the same cell type and time point by calculating the Bhattacharyya distance (86). This measure is used to calculate the amount of overlap in the AUC of the 2 sample distributions (corresponding to each group). BD compares distribution of gene relative to abundance (in TPM). The Bhattacharyya coefficient [BC(Q)i] ranges between 0 (for totally nonoverlapping distributions) and 1 (for completely identical distributions) and is derived from the Bhattacharyya distance. In our analysis, we used a modified form of the Bhattacharyya coefficient that ranges between 0 (for completely identical distributions) and +1 or –1 (for totally nonoverlapping distributions; sign defined by the log-FC value). DE genes were identified if the absolute value of SSMD was greater than or equal to 0.9, the absolute value of BC was greater than or equal to 0.5, and FC was greater than or equal to 1.33. Coding for bioinformatics analysis and data visualization was performed in Python (version 3.7 with Anaconda distribution).

Gene Ontology analysis of DEGs was carried out using Enrichr (https://maayanlab.cloud/Enrichr/) (87, 88).

### scRNA-Seq

The protocol was adopted from previous research (29). Mice were deeply anesthetized and transcardially perfused with ice-cold HBSS. The spinal cords were then extracted using 3 mL HBSS by inserting a 20G needle at the caudal end of the vertebral column. Spinal cords were dissected and placed in a well plate containing 3 mL DMEM with 2 mg/mL collagenase type IV (Gibco). The tissue was minced with scissors and treated with 1 μL DNase I (Thermo Fisher Scientific) for 45 minutes at 37°C.

After 20 minutes of incubation, the tissue was Dounce homogenized 20 times with pestle A with rotation of the pestle. The homogenized cell suspensions were transferred back to the well plate and gently agitated by hand 10 minutes later. The cell suspensions were then passed through a 70 μm prewetted (with DMEM) cell strainer, transferred to a prechilled 50 mL tube, and centrifuged at 22°C for 10 minutes (400*g*, acceleration: 2, brake: 1).

The supernatant was decanted, and the cell pellet was washed with 15–20 mL of 1× HBSS, followed by centrifugation under the same conditions. After decanting the supernatant, the pellet was resuspended in 13 mL of 30% Percoll (MilliporeSigma) diluted in HBSS at room temperature. The 13 mL cell suspension was transferred to a 15 mL tube and overlaid with 2 mL HBSS, then centrifuged at 22°C for 20 minutes (400*g*, acceleration: 1, brake: 0).

The debris and Percoll were carefully removed without disturbing the immune cell pellet at the bottom of the tube. The cell pellet was washed with 15 mL ice-cold HBSS and centrifuged at 4°C for 7 minutes (500*g*, acceleration: 2, brake: 1). After removal of the supernatant, the pellet was resuspended in 500 μL ice-cold FACS buffer (0.4% nonacetylated BSA in 1× PBS, sterile) containing CD11b (PE), CD45 (APC-Cy7), and CX3CR1 antibodies (BioLegend) at a 1:200 dilution. The samples were incubated on ice, protected from light, for 1 hour. The stained samples were washed with 14 mL ice-cold HBSS and centrifuged again at 4°C for 7 minutes (500*g*, acceleration: 2, brake: 1). The supernatant was carefully removed, and the pellet was resuspended in 500 μL ice-cold FACS buffer. The samples were kept on ice and protected from light until sorting. Sorting was performed using either a FACSAria III cell sorter equipped with 405 nm, 488 nm, and 640 nm lasers or a FACSAria Fusion with 405, 488, 561, and 633 nm lasers (both from BD Biosciences). In both cases, live, single CD11b^hi+^, CD45^lo^, and CX3CR1^hi+^ cells were sorted using a 70 μm nozzle at 70 psi. Gating strategies were determined using fluorescence-minus-one controls.

### scRNA-Seq data processing and analysis

Droplet libraries were processed using *Cell Ranger count* pipeline (89). Sequencing reads were aligned to the mm10 mouse reference genome, and transcript counts were quantified for each annotated gene within every cell. Count matrices (genes × cells) were loaded into the R package Seurat (90) for quality control and downstream analyses. Low-quality cells were filtered out using the following criteria: (a) the number of detected genes ≤300; (b) percentage of mitochondrial RNA ≥5% per cell. Cell doublets were detected and removed using the R package *scDblFinder* (91). After filtering, we had 1,014 cells in the WT group and 386 cells in the 4E-BP1–cKO group. Following *SCTransform* normalization, individual samples were integrated using the *RPCAIntegration* procedure. Uniform manifold approximation and projection (UMAP) dimension reduction was generated based on the first 20 principal components. A nearest-neighbor graph using the first 20 principal components was calculated using the *FindNeighbors* function, followed by clustering using the *FindClusters* function with resolution = 1.4. Cluster-specific marker genes were identified using the function *FindMarkers* with the following cutoffs: log_2_ FC > 0.5 and adjusted *P* < 0.05 (upregulated genes only). Per-cluster differential expression testing between the 2 samples was conducted using a single-cell approach with *FindMarkers*. To test for signature enrichment in clusters, we first used the *AddModuleScore* function to calculate the normalized average expression of each marker set in single cells and then performed Wilcoxon’s test to compare the values in cells from designated clusters to all remaining cells.

### Statistics

All data were analyzed using GraphPad Prism v.9 and presented as mean ± SEM. Statistical analyses were performed using 1-way or 2-way ANOVA followed by between-group comparisons using Tukey’s post hoc test or unpaired 2-tailed *t* test. A *P* value less than 0.05 was the significance criterion.

### Study approval

Housing and all experimental procedures on mice complied with the guidelines of the Canadian Council on Animal Care and the International Association for the Study of Pain and were approved by the McGill University Downtown Animal Care Committee.

### Data availability

Sequencing data generated in this study were deposited in the Gene Expression Omnibus database (GEO GSE254472 and GSE285677). Values for all data points in graphs are reported in the Supporting Data Values file.

## Author contributions

KCL, JSM, and AK conceived the project, designed experiments, and supervised the research. KCL, CW, and NB performed behavioral and IHC studies. WC, PS, RR, MH, NFSC, and JF assisted with IHC and microglia analyses. SU, DTF, NNI, KM, and TJP assisted with TRAP analysis. ST, JY, VH, ARS, and CGG assisted with study design and interpretation of results. AP performed the scRNA-Seq bioinformatic analysis. KCL, JSM, and AK wrote the manuscript. All authors reviewed the manuscript and discussed the work.

## Supplementary Material

Supplemental data

Supplemental table 1

Supplemental table 2

Supporting data values

## Version 1 06/02/2025

Electronic publication
