# Supplementary material for: 4E-BP1–dependent translation in microglia controls mechanical hypersensitivity in male and female mice
Source: J Clin Invest. 2025 Jun 2;135(11):e180190. doi: 10.1172/JCI180190 (PMC12126233; doi:10.1172/JCI180190)
Supplement: Supplemental data [file jci-135-180190-s051.pdf]

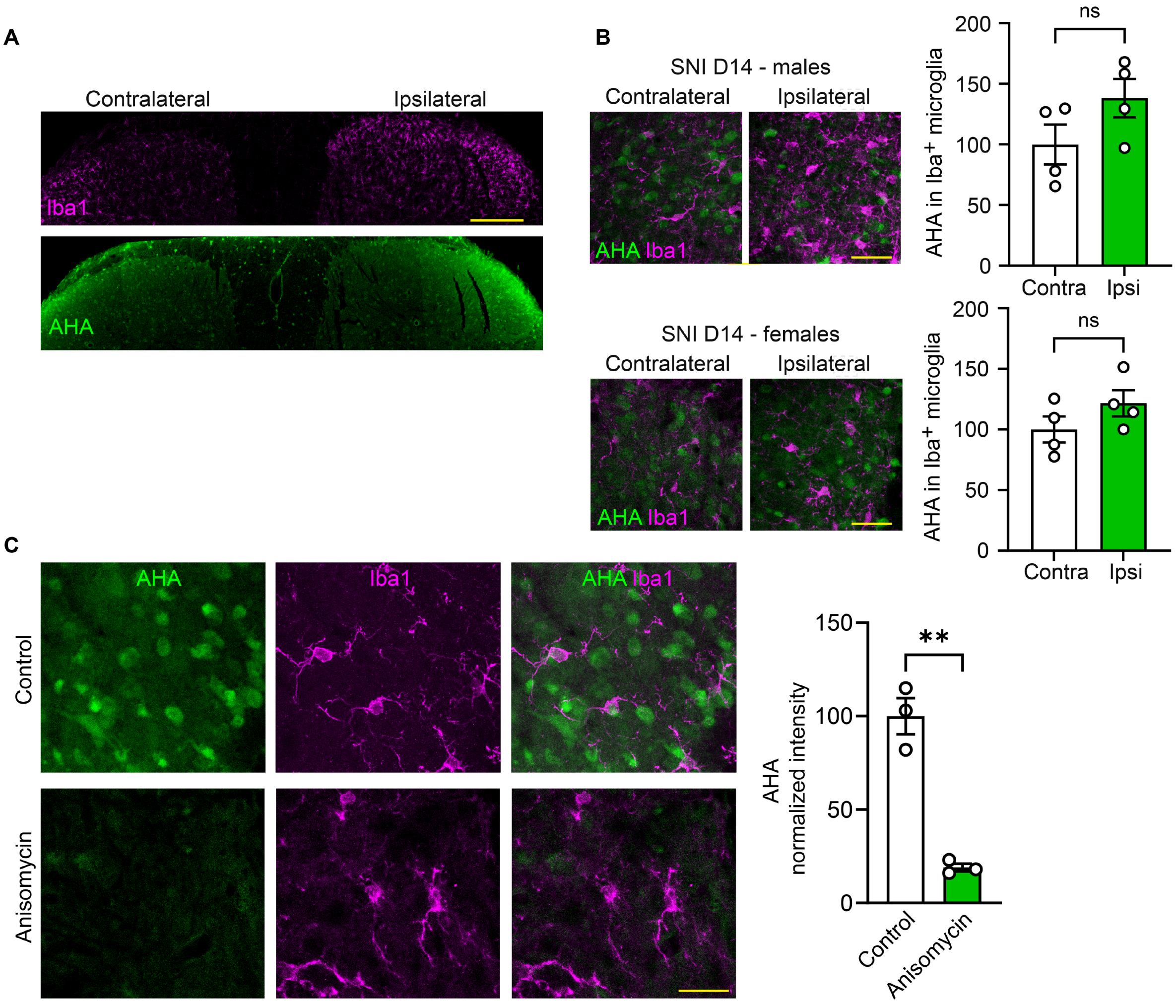


**Supplemental Figure 1. Inhibition of protein synthesis using anisomycin.**

(**A**) Low-magnification images of Iba1 and AHA (FUNCAT) in the spinal cord 4 days post-SNI in male mice (related to Figure 1). Scale bar is 150 µm. (**B**) Assessment of AHA incorporation in male (top) and female (bottom) mice 14 days post-SNI. Quantification (normalized to the contralateral side, n = 4/group) is presented on the right. Scale bar is 30 µm. (**C**) FUNCAT signal (AHA incorporation) was measured in Iba1$^{+}$ microglia in control mice and in mice treated with protein synthesis inhibitor, anisomycin. Representative images show blockade of AHA incorporation in anisomycin-injected mice. Scale bar is 20 µm. Quantification of AHA incorporation in microglia (normalized to Control, n = 3 mice per group) is on the right. An unpaired two-tailed *t*-test. Data are plotted as mean ± s.e.m. $^{**}p < 0.01$,

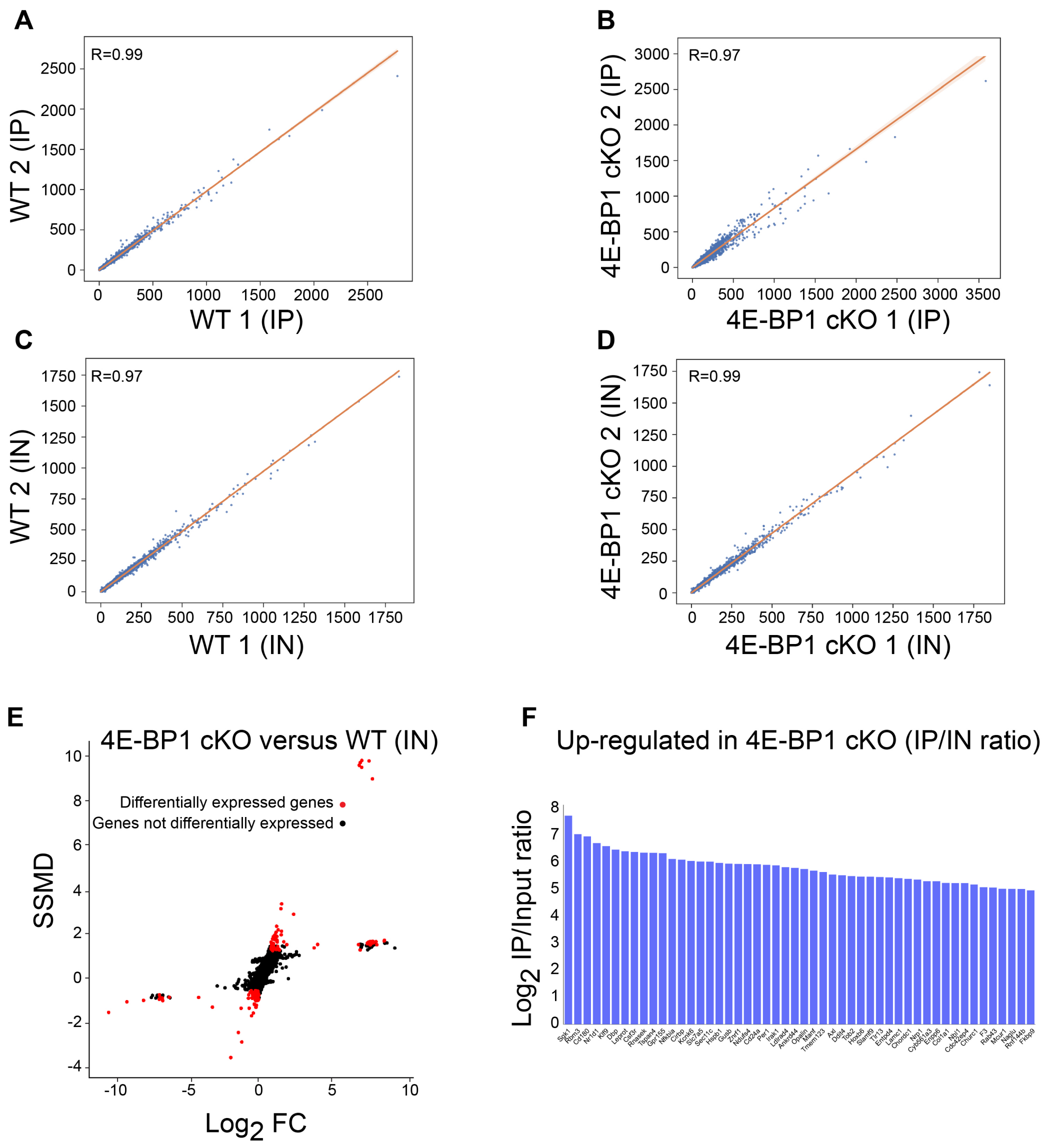


**Supplemental Figure 2. Analysis of TRAP-seq.**

Correlation coefficients of genes between biological replicates for WT (**A**) and 4E-BP1 cKO (**B**) samples in the IP and for WT (**C**) and 4E-BP1 cKO (**D**) in the IN fractions, using linear correlation plots, indicating high reproducibility between experiments. (**E**) Dual-flashlight plot of IN samples showing SSMD versus $\log_2$ FC. (**F**) $\text{Log}_2$ IP/IN ratio of top 50 genes.

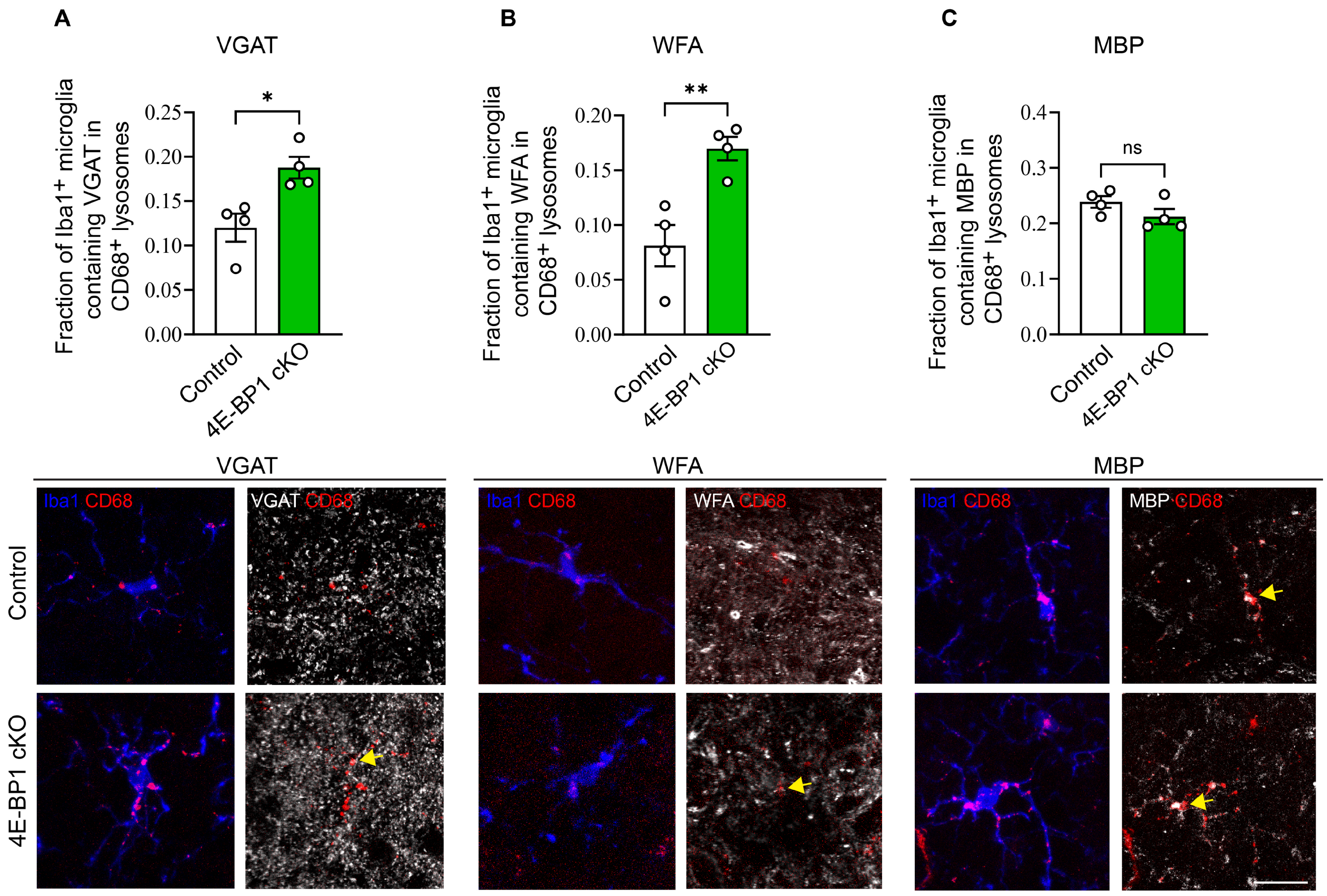


**Supplemental Figure 3. Assessment of microglial lysosomal content in 4E-BP1 cKO mice**

Immunostaining of spinal cord dorsal horn from 4E-BP1 cKO and Control mice for VGAT (vesicular GABA transporter, **A**), WFA (Wisteria floribunda agglutinin, **B**), and MBP (myelin basic protein, **C**), as well as Iba1 and CD68. Scale bar is 15 µm. Quantification of fraction of $Iba1^{+}$ microglia containing VGAT (**A**), WFA (**B**), or MBP (**C**) in $CD68^{+}$ lysosomes is shown at the top (n = 4 mice per group). An unpaired two-tailed *t*-test. Data are plotted as mean ± s.e.m. $^{*}p < 0.05$, $^{**}p < 0.01$, ns – not significant.

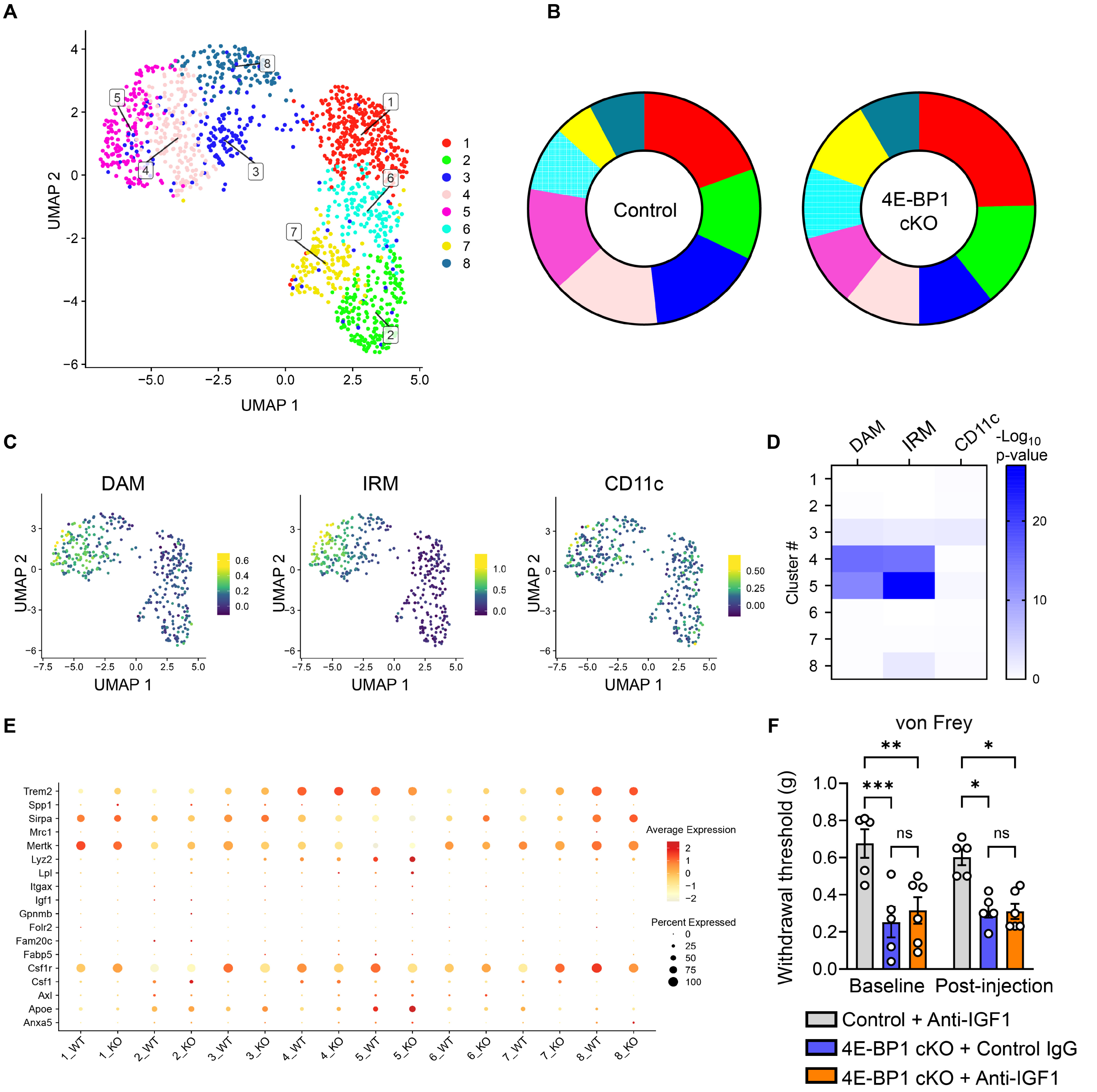


**Supplemental Figure 4. scRNA-seq of spinal microglia from 4E-BP1 cKO mice**

Spinal microglia from 4E-BP1 cKO and Control (*Eif4ebp1*$^{fl/fl}$) mice were sorted (gated for CD45$^{low}$CD11B$^{high}$CX3CR1$^{high}$), captured (10xGenomics NextGem scRNA 3' V3.1,) and sequenced (NovaSeq X Plus 10B, PE100). (**A**) UMAP plot shows that microglia in the mouse spinal cord in both groups are present in 9 distinct clusters. (**B**) The proportion of microglia in each cluster in 4E-BP1 cKO

and Control mice. Color coding corresponds to 8 microglial clusters in **A**. The similarity between cellular transcriptome and previously described transcriptional signatures associated with disease-associated microglia (DAM), injury-responsive microglia (IRM), and $CD11c^{+}$ microglia (full lists of genes used for each signature are in Supplementary Data 2) is shown using UMAP plots (**C**) or heatmap (per each cluster using two-sided Fisher's exact test, **D**). In **D**, white indicates no significant overlap ($p > 0.05$). Shades of blue (for p value $< 0.05$) indicate the significance of the overlap between two gene lists. (**E**) The average expression level and % of expressing cells for distinct transcripts (largely associated with $CD11c^{+}$ microglia) is shown per sample and genotype. (**F**) 4E-BP1 cKO mice were administered intrathecally with the anti-IGF1-neutralizing antibody and normal goat IgG (for control) for three consecutive days. Control (*Eif4ebp1*$^{fl/fl}$) mice were administered with the anti-IGF1-neutralizing antibody. The von-Frey thresholds were assessed before the injection (Baseline) and seven days after the first injection (post-injection). One-way ANOVA followed by Tukey's post-hoc comparison was used in **F**. Data are plotted as mean ± s.e.m. *$p < 0.05$, **$p < 0.01$, ***$p < 0.001$, ns – not significant.

**Supplemental Data 1. Analysis of TRAP-sequencing data. Tab A**: Raw transcripts per million for INPUT and respective percentiles. **Tab B**: Raw transcripts per million for IP and respective percentiles. **Tab C**: Quantile normalized transcripts per million for IP and respective statistics. **Tab D**: Summary of differentially expressed genes between WT and 4E-BP1 cKO in IP samples.

**Supplemental Data 2. Genes associated with the DAM, IRM and CD11c transcriptional signature.**
